# Supplementary material for: AI-augmented intraoperative decision-making workflows in diffuse midline glioma biopsy using cryosection pathology
Source: Nat Commun. 2025 Nov 26;16:11667. doi: 10.1038/s41467-025-66853-y (PMC12749771; doi:10.1038/s41467-025-66853-y)
Supplement: Supplementary file 1 — Supplementary Information [file 41467_2025_66853_MOESM1_ESM.pdf]

Supplementary information

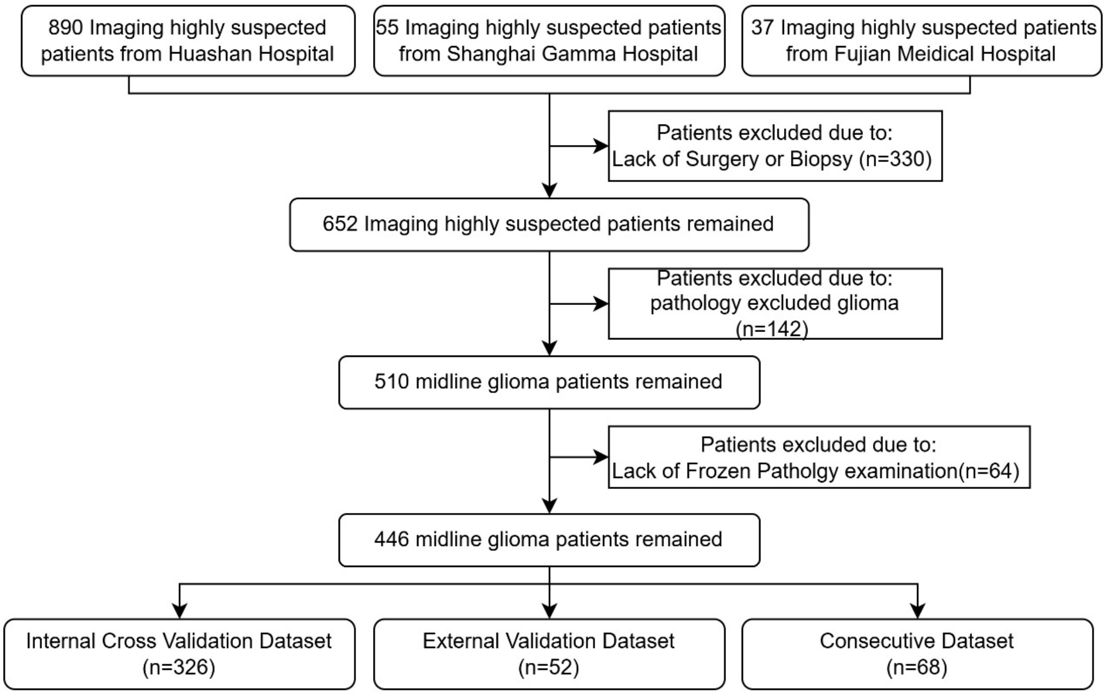

Figure S1. The workflow of data inclusion and exclusion criteria.

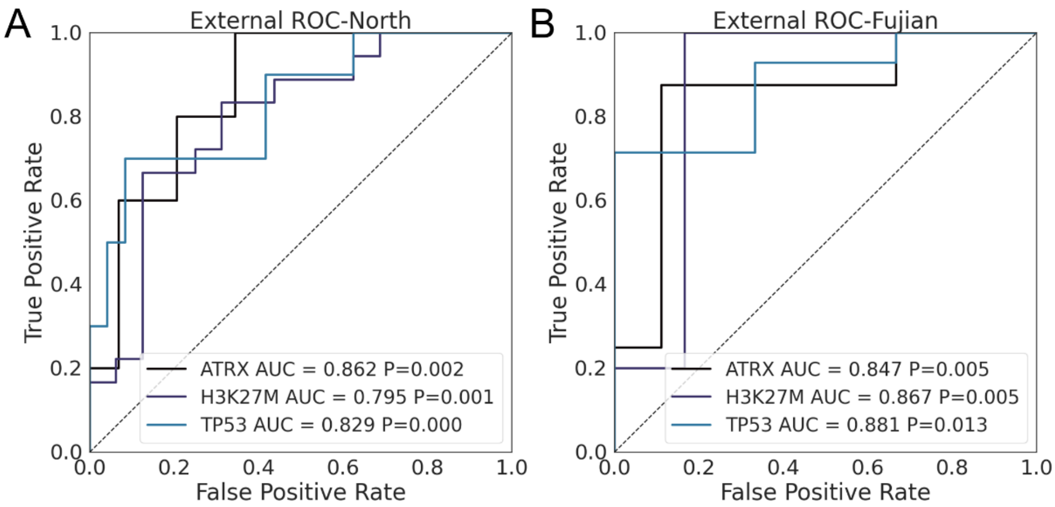

Figure S2. Centre specific ROCs with AUC values for North and Fujian hospital. (A) ROC for North hospital dataset. (B). ROC for Fuujian hospital dataset. Source data are provided as a Source Data file.

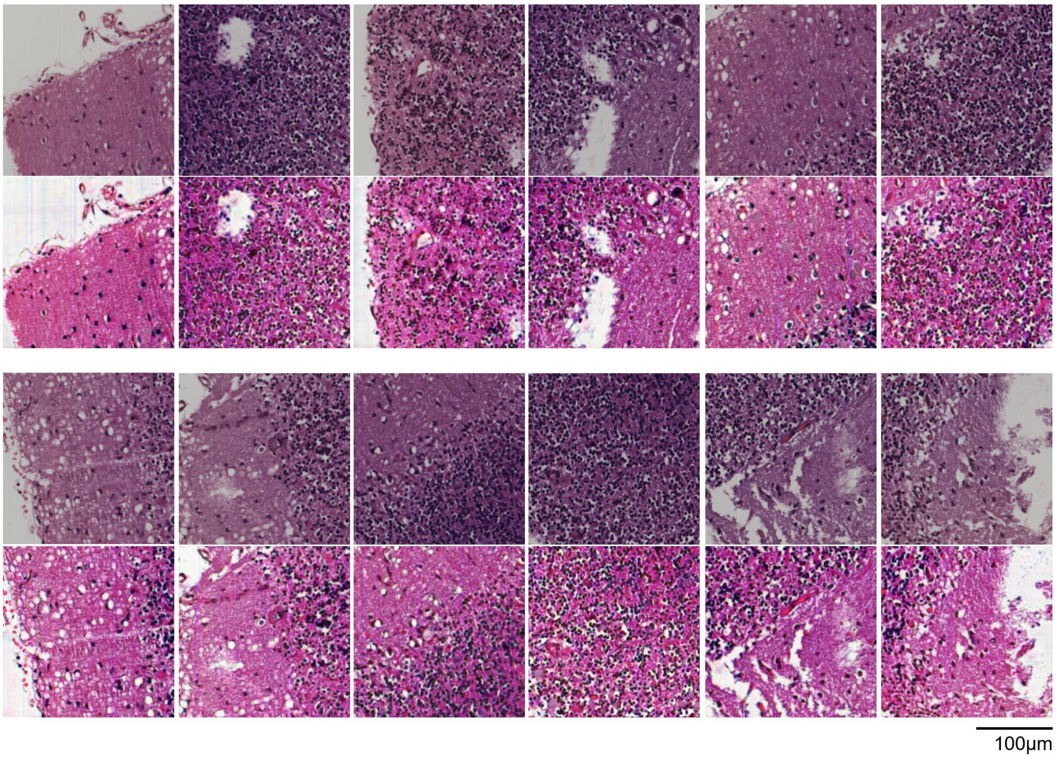

Figure S3. Exemplified cryosection pathology images (1st and 3rd rows) and the corresponding AI-FFPE generated images (2nd and 4th rows).

Table S1. The demographics information and mutant information for the included datasets.

	Internal Cross Validation	Multi-centre External Validation	Consecutive Validation
**Patient Numbers**	327	52	68
**General Information**			
**Age(years)-median(range)**	34 (4-75)	10 (2-70)	39 (5-75)
**Sex-n(%)**			
**Female**	139 (42.5)	23 (44.2)	40 (58.8)
**Male**	188 (57.5)	29 (55.8)	28 (41.2)
**Radiographic Data**			
**Location-n(%)**			
**Thalamic**	180 (55.1)	13 (25.0)	39 (57.4)
**Mesencephalon**	14 (4.3)	3 (5.8)	1 (1.5)
**Pons**	92 (28.1)	28 (53.8)	13 (19.1)
**Medulla oblongata**	34 (10.4)	5 (9.6)	12 (17.6)
**Multiple**	7 (2.1)	3 (5.8)	3 (4.4)
**Side-n(%)**			
**Left**	134 (41.0)	19 (36.5)	25 (36.8)
**Right**	127 (38.8)	15 (28.9)	22 (32.3)
**Both**	66 (20.2)	18 (34.6)	21 (30.9)
**Surgical Procedure-n(%)**			
**Biopsy**	202 (61.8)	26 (50.0)	46 (68.7)
**Resection**	125 (38.2)	26 (50.0)	21 (31.3)
**Histological Data**			
**WHO Grade-n(%)**			
**Grade 1**	39 (11.9)	9 (17.3)	6 (8.8)
**Grade 2**	25 (7.6)	6 (11.5)	11 (16.2)
**Grade 3**	8 (2.5)	3 (5.8)	1 (1.5)
**Grade 4**	255 (78.0)	34 (65.4)	50 (73.5)
**H3K27M-n(%)**			
**mutation**	172 (52.6)	23 (44.2)	33 (48.5)
**ATRX-n(%)**			
**loss**	94 (28.7)	14 (26.9)	26 (38.2)
**TP53-n(%)**			
**missense mutation**	129 (39.4)	24 (46.2)	31 (45.6)
**IDH-n(%)**			
**mutation**	14 (4.3)	7 (13.5)	8 (11.8)

Table S2. Performance metrics of CryoAID given once and twice biopsies in retrospective re-checking using the internal dataset.

	Gene	ACC	F1	SEN	SPE	AUC
**Once**	**ATRX**	0.681±0.07	0.549±0.12	0.600±0.08	0.708±0.08	0.722±0.07
	**H3K27M**	0.675±0.08	0.767±0.04	0.646±0.08	0.711±0.09	0.740±0.07
	**TP53**	0.733±0.08	0.729±0.07	0.681±0.07	0.760±0.09	0.781±0.08
**Twice**	**ATRX**	0.680±0.13	0.645±0.14	0.602±0.11	0.720±0.15	0.777±0.10
	**H3K27M**	0.718±0.11	0.807±0.11	0.688±0.11	0.761±0.13	0.792±0.14
	**TP53**	0.775±0.06	0.817±0.09	0.681±0.07	0.838±0.09	0.844±0.07
