# Supplementary material for: AI-augmented intraoperative decision-making workflows in diffuse midline glioma biopsy using cryosection pathology
Source: Nat Commun. 2025 Nov 26;16:11667. doi: 10.1038/s41467-025-66853-y (PMC12749771; doi:10.1038/s41467-025-66853-y)
Supplement: Supplementary file 3 — Reporting Summary [file 41467_2025_66853_MOESM3_ESM.pdf]

nature portfolio

Corresponding author(s): Mianxin Liu

Last updated by author(s): Oct 13, 2025

Reporting Summary

Nature Portfolio wishes to improve the reproducibility of the work that we publish. This form provides structure for consistency and transparency in reporting. For further information on Nature Portfolio policies, see our Editorial Policies and the Editorial Policy Checklist.

Statistics

For all statistical analyses, confirm that the following items are present in the figure legend, table legend, main text, or Methods section.

n/a	Confirmed	
☐	☒	The exact sample size (*n*) for each experimental group/condition, given as a discrete number and unit of measurement
☐	☒	A statement on whether measurements were taken from distinct samples or whether the same sample was measured repeatedly
☐	☒	The statistical test(s) used AND whether they are one- or two-sided<br>*Only common tests should be described solely by name; describe more complex techniques in the Methods section.*
☒	☐	A description of all covariates tested
☒	☐	A description of any assumptions or corrections, such as tests of normality and adjustment for multiple comparisons
☐	☒	A full description of the statistical parameters including central tendency (e.g. means) or other basic estimates (e.g. regression coefficient) AND variation (e.g. standard deviation) or associated estimates of uncertainty (e.g. confidence intervals)
☐	☒	For null hypothesis testing, the test statistic (e.g. *F*, *t*, *r*) with confidence intervals, effect sizes, degrees of freedom and *P* value noted<br>*Give P values as exact values whenever suitable.*
☒	☐	For Bayesian analysis, information on the choice of priors and Markov chain Monte Carlo settings
☒	☐	For hierarchical and complex designs, identification of the appropriate level for tests and full reporting of outcomes
☒	☐	Estimates of effect sizes (e.g. Cohen's *d*, Pearson's *r*), indicating how they were calculated

Our web collection on statistics for biologists contains articles on many of the points above.

Software and code

Policy information about availability of computer code

Data collection	No software was used.
Data analysis	We used Python 3.10 and Pytorch 2.2.2. For previously published models and methods: The model for AI-FFPE is available at https://github.com/DeepMIALab/AI-FFPE. CHIEF model is available at https://github.com/hms-dbmi/CHIEF. UNI model is available at https://github.com/mahmoodlab/UNI. Gigapath model is available at https://github.com/prov-gigapath/prov-gigapath. Virchow2 model is available at https://huggingface.co/paige-ai/Virchow2. Pathoduet model is available at https://github.com/openmedlab/PathoDuet. The code for ABMIL is available at https://github.com/AMLab-Amsterdam/AttentionDeepMIL. The code for CLAM and the ResNet model is available at https://github.com/mahmoodlab/CLAM. The code for TransMIL is available at https://github.com/szc19990412/TransMIL. Our self-developed codes are documented at https://github.com/MianxinLiu/CryoAID (DOI: 10.5281/zenodo.17393827)44 and released under MIT license.

For manuscripts utilizing custom algorithms or software that are central to the research but not yet described in published literature, software must be made available to editors and reviewers. We strongly encourage code deposition in a community repository (e.g. GitHub). See the Nature Portfolio guidelines for submitting code & software for further information.

Data

Policy information about availability of data

All manuscripts must include a data availability statement. This statement should provide the following information, where applicable:

- Accession codes, unique identifiers, or web links for publicly available datasets
- A description of any restrictions on data availability
- For clinical datasets or third party data, please ensure that the statement adheres to our policy

The multi-centre clinical dataset (extracted pathology image feature and gene mutant label, being anonymous, together with trained model and data splits) generated in this study have been deposited in the Zenodo database under accession code 17373753 [DOI: 10.5281/zenodo.17373753]. The data are available under restricted access for the privacy protection restriction of hospital, access can be obtained by sending online request via Zenodo. The request will be reviewed online and responded within one weak. The approval will be given based on the reasonableness of the research purposes of the request, and commercial usage is strictly forbidden. Source data are provided with this paper.

Research involving human participants, their data, or biological material

Policy information about studies with human participants or human data. See also policy information about sex, gender (identity/presentation), and sexual orientation and race, ethnicity and racism.

Reporting on sex and gender	We follow the 'Sex and Gender Equity in Research – SAGER – guidelines' and to include sex and gender considerations where relevant. The study is designed based on biological attribute. Sex was self-reported by participants and included in the demographics information analysis, as additional information in Supplementary Table 1.
Reporting on race, ethnicity, or other socially relevant groupings	All participants are local Han Chinese.
Population characteristics	General information, radiographic Data, surgical procedure, histological data, and genotypic information
Recruitment	Participants were retrospectively recruited from patients with midline glioma who had undergone surgery in Huashan Hospital, North Campus of Huashan Hospital, Huashan Hospital Fujian Campus and Shanghai Gamma Hospital (with the participation of neurosurgeons and pathologists from Huashan Hospital). There is no potential self-selection bias or other bias.
Ethics oversight	This research was approved by the Institutional Review Board of Huashan Hospital (KY2024-1242). Patients have signed informed consent forms for enrolling in CNS disease bank in advance of operations, authorizing to use their pathological images for this study.

Note that full information on the approval of the study protocol must also be provided in the manuscript.

Field-specific reporting

Please select the one below that is the best fit for your research. If you are not sure, read the appropriate sections before making your selection.

☒ Life sciences ☐ Behavioural & social sciences ☐ Ecological, evolutionary & environmental sciences

For a reference copy of the document with all sections, see nature.com/documents/nr-reporting-summary-flat.pdf

Life sciences study design

All studies must disclose on these points even when the disclosure is negative.

Sample size	447 patients were included. No calculation is performed. DMG is rare disease and this sample size is comparable to the published DMG works.
Data exclusions	Data exclusion criteria includes lack of frozen patholgy examination, lack of surgery or biopsy, pathology exclusion. Workflow of data inclusion and exclusion criteria is described in Figure S1.
Replication	This study is a multi-center retrospective study, and the stability of the model was replicated across different centers.
Randomization	This study is a multi-center retrospective study, and the classification criteria of different datasets are determined by different centers and enrollment periods, thus this study was not randomized.
Blinding	This study is a retrospective study and is not blinded.

Reporting for specific materials, systems and methods

We require information from authors about some types of materials, experimental systems and methods used in many studies. Here, indicate whether each material, system or method listed is relevant to your study. If you are not sure if a list item applies to your research, read the appropriate section before selecting a response.

Materials & experimental systems

n/a	Involved in the study
☒	☐ Antibodies
☒	☐ Eukaryotic cell lines
☒	☐ Palaeontology and archaeology
☒	☐ Animals and other organisms
☒	☐ Clinical data
☒	☐ Dual use research of concern
☒	☐ Plants

Methods

n/a	Involved in the study
☒	☐ ChIP-seq
☒	☐ Flow cytometry
☒	☐ MRI-based neuroimaging

Plants

Seed stocks	*Report on the source of all seed stocks or other plant material used. If applicable, state the seed stock centre and catalogue number. If plant specimens were collected from the field, describe the collection location, date and sampling procedures.*
Novel plant genotypes	*Describe the methods by which all novel plant genotypes were produced. This includes those generated by transgenic approaches, gene editing, chemical/radiation-based mutagenesis and hybridization. For transgenic lines, describe the transformation method, the number of independent lines analyzed and the generation upon which experiments were performed. For gene-edited lines, describe the editor used, the endogenous sequence targeted for editing, the targeting guide RNA sequence (if applicable) and how the editor was applied.*
Authentication	*Describe any authentication procedures for each seed stock used or novel genotype generated. Describe any experiments used to assess the effect of a mutation and, where applicable, how potential secondary effects (e.g. second site T-DNA insertions, mosiacism, off-target gene editing) were examined.*
